# Oxidation-Active Radical TTM-DMODPA for Catalysis-Free Hydrogen Peroxide Colorimetric Sensing

**DOI:** 10.3390/bios15080490

**Published:** 2025-07-29

**Authors:** Qingmei Zhong, Xiaomei Rong, Tingting Wu, Chuan Yan

**Affiliations:** Hunan Engineering Research Center for Monitoring and Treatment of Heavy Metals Pollution in the Upper Reaches of Xiangjiang River, College of Chemistry and Materials Science, Hengyang Normal University, Hengyang 421008, China

**Keywords:** visual determination, hydrogen peroxide, radical TTM-DMODPA, non-catalytic, instrument-free, smartphone camera

## Abstract

As a crucial reactive oxygen species, hydrogen peroxide (H_2_O_2_) serves as both a physiological regulator and a pathological indicator in human systems. Its urinary concentration has emerged as a valuable biomarker for assessing metabolic disorders and renal function. While conventional colorimetric determination methods predominantly employ enzymatic or nanozyme catalysts, we present an innovative non-catalytic approach utilizing the redox-responsive properties of organic neutral radicals. Specifically, we designed and synthesized a novel radical TTM-DMODPA based on the tris (2,4,6-trichlorophenyl) methyl (TTM) scaffold, which exhibits remarkable optical tunability and oxidative sensitivity. This system enables dual-mode H_2_O_2_ quantification: (1) UV-vis spectrophotometry (linear range: 2.5–250 μmol/L, LOD: 1.275 μmol/L) and (2) smartphone-based visual analysis (linear range: 2.5–250 μmol/L, LOD: 3.633 μmol/L), the latter being particularly suitable for point-of-care testing. Validation studies using urine samples demonstrated excellent recovery rates (96–104%), confirming the method’s reliability for real-sample applications. Our work establishes a portable, instrument-free platform for urinary H_2_O_2_ determination, with significant potential in clinical diagnostics and environmental monitoring.

## 1. Introduction

The concentration of hydrogen peroxide (H_2_O_2_) in urine has emerged as a potential biomarker for assessing both the redox homeostasis and microbial ecology within the urogenital tract [1,2,3,4,5,6]. Elevated or depleted urinary H_2_O_2_ levels may indicate pathological conditions, including but not limited to urinary tract infections and systemic metabolic dysregulation, owing to its dual role in antimicrobial defense and oxidative stress modulation [7,8,9,10].

Numerous analytical techniques have been developed for H_2_O_2_ determination, encompassing electrochemical [11,12,13,14,15], chromatographic [16], enzymatic [17,18], chemiluminescence [19], UV-vis [20], and fluorescence spectroscopic methods [21,22]. Among these, the colorimetric method has emerged as the technique of choice for routine analysis, owing to its operational simplicity, cost-effectiveness, and high-throughput capability, particularly in resource-limited settings [23,24,25,26,27]. Presently, the majority of H_2_O_2_ colorimetric assays rely on the TMB (3,3′,5,5′-tetramethylbenzidine) chromogenic reaction [25,26,28,29,30,31,32,33,34], necessitating the involvement of enzymes or enzyme mimics that inevitably complicate the sensing system. Notably, to the best of our knowledge, no non-catalytic colorimetric approach utilizing organic radicals has been reported for H_2_O_2_ determination.

Stable organic radicals exhibit unique electronic properties and a positive response to external oxidation stimulus originating from the intramolecular unpaired electrons [35,36,37]. Among reported organic radicals, tris (2,4,6-trichlorophenyl)methyl (TTM) radical derivatives possess excellent stability due to the effective steric hindrance around the center carbon radical [38,39,40]. Compared with other open-shell radicals, arylamine-substituted TTM radicals have a low band gap and fast conversion from a neutral state to a cation state under the applied electronic field or oxidation species because of the effective intramolecular charge transfer (ICT) interactions [41,42]. This provides accessibility to utilizing a TTM radical positive response to an oxidation stimulus to realize oxidation species determination.

**Scheme 1:**
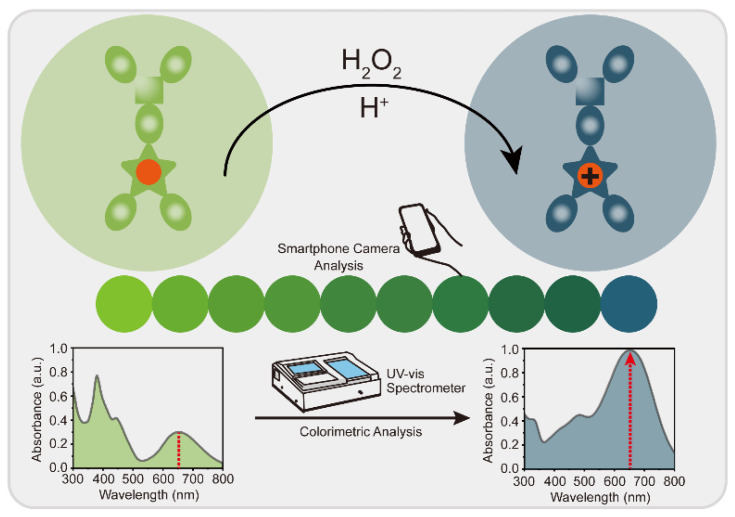
Illustration for hydrogen peroxide visual determination.

In this study, we successfully synthesized a novel organic neutral radical, specifically the 4,4′-dimethoxydiphenylamine-substituted tris(2,4,6-trichlorophenyl)methyl radical (radical TTM-DMODPA). The oxidation process of radical TTM-DMODPA was found to be precisely regulated by H_2_O_2_, resulting in both a measurable increase in absorbance and a distinct colorimetric transition from grass green to blue (Scheme 1). Using UV-vis spectrophotometry, we established a well-defined linear correlation between H_2_O_2_ concentrations and corresponding absorbance changes. Furthermore, we successfully developed a parallel quantitative relationship between H_2_O_2_ concentrations and RGB color values through smartphone-based image analysis. This work presents a significant advancement in analytical methodology: a non-enzymatic, catalyst-free visual determination system for H_2_O_2_ that has been successfully validated in real urine samples. The developed approach offers promising potential for innovative applications in biological sensing and clinical diagnostics. Building upon the investigations of TTM derivatives in optoelectronic fields, this study represents an entirely novel research direction by strategically purposing these radical species for biomedical sensing applications [42,43]. Distinct from the established focus on OLEDs and photothermal conversion, we engineered a TTM-based platform that achieves catalyst-free H_2_O_2_ quantification through radical-specific chromogenic reactions, enabling dual-mode determination (UV-vis: 1.275 μmol/L LOD; smartphone: 3.633 μmol/L LOD). This breakthrough not only circumvents the stability limitations of enzymatic/metal catalysts but also demonstrates exceptional recovery in complex biofluids. By bridging the gap between organic radical chemistry and point-of-care diagnostics, our study establishes a new paradigm for TTM materials beyond their traditional optoelectronic roles, with direct implications for non-invasive disease monitoring and portable healthcare devices.

## 2. Materials and Methods

### 2.1. Chemicals and Reagents

All chemical reagents were obtained from commercial sources and used without further purification. Hydrochloric acid (HCl, 36–38%), hydrogen peroxide (H_2_O_2_, 30%), chloroform, sodium hypochlorite (NaClO), and toluene were purchased from Sinopharm Chemical Reagent Co., Ltd. (Shanghai, China). 1,3,5-trichlorobenzene, aluminium chloride (AlCl_3_), 4,4′-dimethoxydiphenylamine, tetrahydrofuran (THF), potassium tert-butoxide (t-BuOK), and *p*-chloranil were acquired from Anhui Senrise Technology Co., Ltd. (Hefei, China). Catalytic reagents bis(dibenzylideneacetone)palladium (Pd(dba)_2_), 2,2′-bis(diphenylphosphino)-1,1′-binaphthalene (BINAP), sodium tert-butoxide (*t*-BuONa), and ferrous chloride tetrahydrate (FeCl_2_•4H_2_O), xanthine, and xanthine oxidase were sourced from Shanghai Titan Scientific Co., Ltd. (Shanghai, China). Analytical grade inorganic salts (KCl, NaCl, CaCl_2_, trisodium phosphate) and biological compounds (glucose, glycine, methionine, proline, histidine, uric acid, urea, cysteine) were obtained from Adamas Reagent Co., Ltd. (Shanghai, China).

### 2.2. Equipment and Characterization

All nuclear magnetic resonance (NMR) spectra were acquired using a Bruker Avance III HD spectrometer (Bruker Corporation, Billerica, MA, USA) operating at 500 MHz for ^1^H NMR and 125 MHz for ^13^C NMR measurements. The following abbreviations are used to describe signal splitting patterns: s (singlet), d (doublet), and m (multiplet). High-resolution mass spectrometry (HRMS) data were obtained using a Thermo Orbitrap Exploris 120 mass spectrometer (Thermo Fisher Scientific, Waltham, MA, USA). Electron paramagnetic resonance (EPR) spectroscopy of radical species was performed at ambient temperature using a Bruker EMXPlus spectrometer (Bruker Corporation, Billerica, MA, USA). UV-vis absorption spectra were recorded on a 754PC spectrophotometer (Yixin Scientific Instrument Co., Shanghai, China).

### 2.3. Synthesis of Compound HTTM-DMODPA

Compound HTTM was synthesized following literature procedures [44].

The target compound HTTM-DMODPA was prepared through a Buchwald–Hartwig coupling reaction under inert conditions [44]. In a nitrogen-filled Schlenk tube, a reaction mixture containing HTTM (0.55 g, 0.99 mmol), 4,4′-dimethoxydiphenylamine (0.14 g, 0.59 mmol), Pd(dba)_2_ (0.10 g, 0.17 mmol), BINAP (0.11 g, 0.18 mmol), *t*-BuONa (0.29 g, 3.02 mmol), and anhydrous toluene (15 mL) was heated to 110 °C with vigorous stirring for 16 h. After cooling to room temperature, the reaction mixture was concentrated under reduced pressure, and the crude product was purified by silica gel column chromatography using a dichloromethane/petroleum ether (1:7 *v*/*v*) as eluent. The desired product HTTM-DMODPA was isolated as a white solid with a 42% yield (0.19 g).

### 2.4. Synthesis of Radical TTM-DMODPA

The transformation was carried out under strict oxygen-free and light-free conditions. A reaction flask charged with HTTM-DMODPA (0.08 g, 0.11 mmol), *t*-BuOK (0.49 g, 4.40 mmol), and anhydrous tetrahydrofuran (12 mL) was purged with nitrogen and stirred at an ambient temperature for 40 h. Subsequently, *p*-chloranil (0.54 g, 2.20 mmol) was added, and the resulting mixture was stirred for an additional 24 h. After completion, the reaction was concentrated in vacuo, and the residue was purified by flash chromatography on silica gel using a dichloromethane/petroleum ether mixture (1:9 *v*/*v*) as eluent. The target radial TTM-DMODPA was isolated as a dark green solid with a 51% yield (0.04 g).

### 2.5. Procedures for H_2_O_2_ Assay

The test solutions were prepared by sequentially mixing 40 μL radical TTM-DMODPA (640 μM), 7.5 μL HCl (8 mM), and 343 μL acetonitrile, followed by the addition of 10 μL H_2_O_2_ at varying concentrations. The reaction mixtures were incubated at 60 °C for 5 min, after which UV-vis spectra and images were acquired. Absorbance changes at 652 nm (Δ*A*) were calculated as Δ*A* = *A* − *A*_0_, where *A* and *A*_0_ represent the absorbance values of the determination system in the presence and absence of H_2_O_2_, respectively. These Δ*A* values served as the dependent variable for constructing the standard curve and optimizing determination parameters. All measurements were performed in triplicate to ensure data reproducibility.

### 2.6. Sample Preparation

The original urine samples were sourced from healthy adult volunteers in our research team under strict anonymization protocols to ensure donor confidentiality, with immediate post-collection refrigeration at 4 °C to maintain sample integrity. For analytical processing, a standardized 10-fold dilution with phosphate buffer (pH 7.4) was implemented, which simultaneously stabilizes H_2_O_2_ by reducing degradation from redox/pH effects, minimizes matrix interference with 96–104% recovery rates, and maintains consistency with literature-standard 5–100× dilution protocols for urinary biomarkers [45,46].

## 3. Results and Discussion

### 3.1. Synthesis and Characterization of the Radical TTM-DMODPA

**Figure 1:**
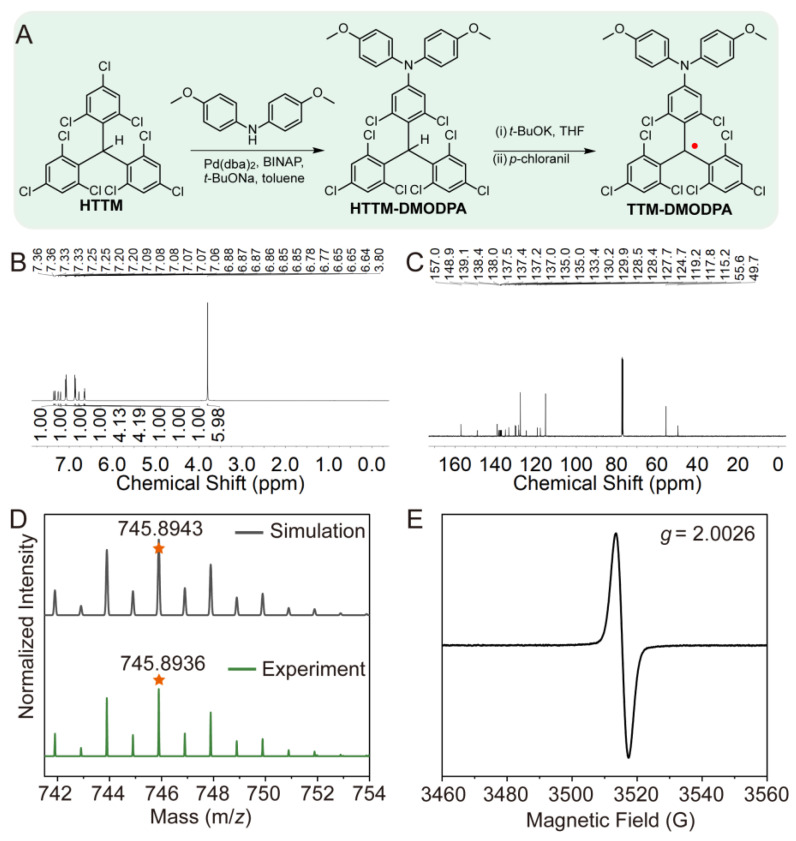
Radical TTM-DMODPA: (**A**) Synthetic route, (**B**) ^1^H NMR spectrum of HTTM-DMODPA in CDCl3. (**C**) ^13^C NMR spectrum of HTTM-DMODPA in CDCl3. (**D**) High resolution mass spectra (HRMS) of simulation and experiment, and (**E**) Electron paramagnetic resonance (EPR).

**Figure 2:**
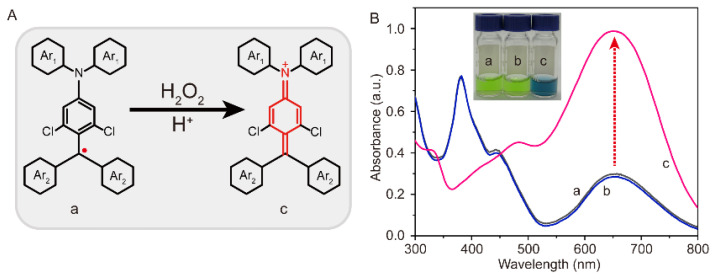
(**A**) Reaction diagram of H_2_O_2_ with radical TTM-DMODPA. (**B**) The UV-vis absorption spectra of different systems. Insert: the corresponding photograph. (a: radical TTM-DMODPA. b: radical TTM-DMODPA/H^+^. c: radical TTM-DMODPA/H^+^/H_2_O_2_).

The target radical TTM-DMODPA was synthesized through a two-step procedure involving dehydrogenation followed by oxidation of its precursor HTTM-DMODPA (Scheme S1). The precursor itself was prepared via a palladium-catalyzed C-N coupling reaction between HTTM and 4,4′-dimethoxydiphenylamine (Figure 1A), which was confirmed by nuclear magnetic resonance (NMR) spectra (collected and processed by TopSpin 4.5.0 software provided with the Bruker Avance III HD spectrometer) (Figure 1B,C, Figures S1 and S2). The assignments of hydrogen and carbon atoms in HTTM-DMODPA are as follows: ^1^H NMR (500 MHz, CDCl_3_) δ (ppm): 7.36 (d, *J* = 2.5 Hz, 1H), 7.33 (d, *J* = 2.0 Hz, 1H), 7.25 (d, *J* = 2.5 Hz, 1H), 7.20 (d, *J* = 2.5 Hz, 1H), 7.09–7.06 (m, 4H), 6.88–6.85 (m, 4H), 6.78 (d, *J* = 2.5 Hz, 1H), 6.65 (d, *J* = 2.5 Hz, 1H), 6.64 (s, 1H), and 3.80 (s, 6H). ^13^C NMR (125 MHz, CDCl_3_) δ (ppm): 157.0, 148.9, 139.1, 138.4, 138.0, 137.5, 137.4, 137.2, 137.0, 135.0, 135.0, 133.4, 130.2, 129.9, 128.5, 128.4, 127.7, 124.7, 119.2, 117.8, 115.2, 55.6, and 49.7. The NMR signals of hydrogen and carbon atoms were consistent with the corresponding molecular structure, which verified the successful synthesis of HTTM-DMODPA. Comprehensive characterization confirmed the successful synthesis of the radical species. High-resolution mass spectrometry (HRMS) analysis revealed a molecular ion peak at *m*/*z* 745.8936 (calcd. 745.8943), and the peaks adjacent to it originate from the isotopic peaks of carbon (^12^C, ^13^C) and chlorine (^35^Cl, ^37^Cl) atoms in the chemical structure (Figure 1D and Figure S3). The radical nature of TTM-DMODPA was unambiguously confirmed by electron paramagnetic resonance (EPR) spectra, which exhibited a characteristic signal at g = 2.0026 [47] (Figure 1E), indicating an open-shell structure with the unpaired electron. UV-vis spectroscopic analysis (Figure 2B(a)) displayed a prominent absorption maximum at 381 nm, corresponding to π-π* transitions characteristic of the triarylmethyl radical family [48]. These collective data provide conclusive evidence for the successful synthesis and structural verification of the target radical TTM-DMODPA.

### 3.2. Feasibility Analysis

The oxidative response of the radical TTM-DMODPA to H_2_O_2_ was systematically investigated. UV-vis spectroscopic analysis (Figure 2B) revealed that upon addition of H_2_O_2_, the characteristic radical absorption at 381 nm exhibited a progressive decrease in absorbance from 0.77 to 0.25. In the visible region, the radical displayed a maximum absorption wavelength at 652 nm. Subsequent introduction of H_2_O_2_ resulted in a pronounced absorbance enhancement at this wavelength, increasing from 0.32 to 0.99, corresponding to a 3.09-fold amplification. This spectral evolution was accompanied by a distinct colorimetric transition from grass green to deep blue, consistent with the formation of quinoid oxidation products (Figure 2A). The isosbestic point at 452 nm (Figure 2B) reflects the dynamic equilibrium between the radical TTM-DMODPA (compound a) and oxidized (compound c) forms of our system, with the constant absorbance (A = 0.395) at this wavelength serving as a built-in quality control parameter. It enhances measurement reliability by providing an internal reference: the invariant absorbance at the isosbestic point enables real-time correction for probe concentration variations (e.g., due to pipetting errors or sample matrix effects) [41]. Collectively, these spectroscopic and visual changes demonstrated the potential of radical TTM-DMODPA as a sensitive colorimetric probe for H_2_O_2_ determination.

### 3.3. Colorimetric Determination of H_2_O_2_

**Figure 3:**
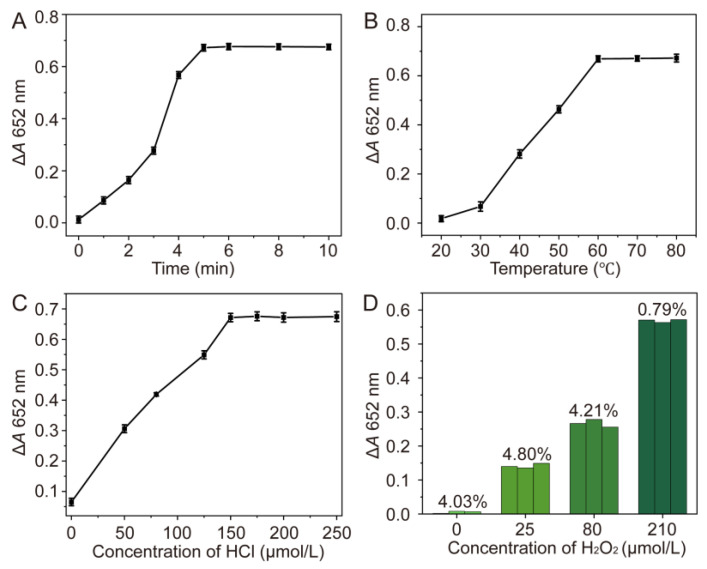
The optimizations of experimental conditions. Effect of (**A**) reaction time, (**B**) reaction temperature, and (**C**) concentration of HCl on the absorbance change at 652 nm. (**D**) Histogram of Δ*A* intensity with the concentration of H_2_O_2_ = 0, 25, 80, 210 μmol/L.

Building upon these findings, we proceeded to systematically optimize and validate the radical TTM-DMODPA oxidation assay for H_2_O_2_ determination. To establish optimal sensing conditions, we investigated three critical parameters: reaction time, temperature, and HCl concentration. First, the reaction time was examined by monitoring absorbance changes (Δ*A*) at 652 nm over time (Figure 3A). The radical solution containing TTM-DMODPA, H_2_O_2_, and HCl was incubated at 60 °C, revealing a rapid Δ*A* increase within the first 5 min followed by signal stabilization, suggesting complete reaction within this timeframe. Temperature optimization studies (Figure 3B) demonstrated progressive Δ*A* enhancement from 20 to 60 °C, with maximal and stable response achieved at 60 °C, which was consequently selected for all subsequent experiments. Finally, HCl concentration was found to significantly influence signal intensity, with Δ*A* showing a concentration-dependent increase up to 150 μmol/L (Figure 3C), beyond which the response plateaued. This optimal HCl concentration was therefore adopted for the developed sensing protocol. Additionally, the radical TTM-DMODPA system is designed for point-of-care testing of ex vivo urine analysis in resource-limited settings, with key advantages including instrument-free, cost-effective, and visual readout capability features specifically optimized for point-of-care testing in community clinics and home use scenarios. The in vitro radical TTM-DMODPA system’s experimental flexibility allows comprehensive parameter optimization beyond physiological constraints, while subsequent validation with pH 4–9 buffer systems (Figure S4) confirmed maintained detection efficiency across the full physiological pH.

**Figure 4:**
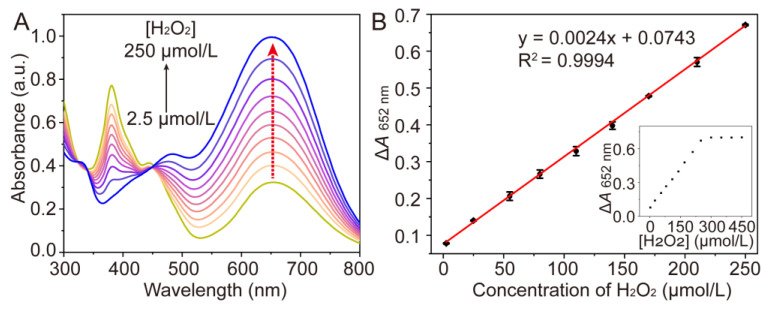
(**A**) UV-vis absorption spectra in the presence of various H_2_O_2_ concentrations. (**B**) The linear relationship between Δ*A*_652 nm_ and H_2_O_2_ concentration.

Following the optimization of experimental parameters, we thoroughly assessed the analytical performance of the method by evaluating its reproducibility, linear dynamic range, determination limit, and selectivity. The radical TTM-DMODPA demonstrated excellent method reproducibility, as evidenced by consistently low relative standard deviations (RSD ≤ 4.80%) across multiple H_2_O_2_ concentration levels (Figure 4). This robust precision profile confirms the reliability of our developed assay for quantitative applications.

The quantitative analytical performance of the radical TTM-DMODPA-based determination system was systematically evaluated. Spectrophotometric analysis (Figure 4A) revealed a concentration-dependent absorbance enhancement at 652 nm proportional to H_2_O_2_ concentration, establishing Δ*A*_652 nm_ as a reliable quantitative indicator. A linear calibration curve was obtained over the concentration range of 2.5–250 μmol/L (Figure 4B), with the regression equation Δ*A*_652 nm_ = 0.0024*C*_H2O2_ + 0.0743 (R^2^ = 0.9994), demonstrating excellent correlation. The method exhibited high sensitivity with a calculated detection limit of 1.275 μmol/L (3σ/slope, where σ represents the standard deviation of 15 blank measurements). Comparative analysis with existing H_2_O_2_ determination methods (Table 1) confirmed that our radical TTM-DMODPA-based approach offered competitive or superior performance in terms of both linear range and determination sensitivity. Notably, the assay requires only radical TTM-DMODPA and acidic conditions (H^+^), eliminating the need for metal catalysts or precious metal components. This simplified reaction system provides distinct advantages in terms of operational simplicity and environmental friendliness. Collectively, these results establish radical TTM-DMODPA as an exceptional radical probe for sensitive and selective H_2_O_2_ quantification via UV-vis spectroscopy.

**Figure 5:**
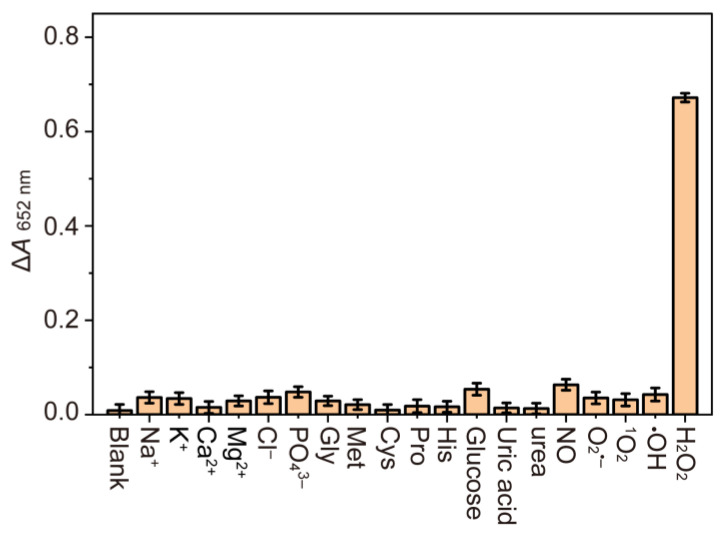
The selectivity of radical TTM-DMODPA system to H_2_O_2_ (H_2_O_2_ was 250 μmol/L, reactive oxygen species were 250 μmol/L, and other substances were 2.5 mmol/L).

To assess the practical applicability of the radical TTM-DMODPA based determination system, we rigorously evaluated its anti-interference capability against 18 potentially competing substances, including biologically relevant ions (Na^+^, K^+^, Ca^2+^, Mg^2+^, Cl^−^, and PO_4_^3−^), amino acids (Gly, Met, Cys, Pro, and His), metabolites (glucose, uric acid, urea, NO), and reactive oxygen species (O_2_^•−^, ^1^O_2_, and •OH) [59]. As clearly demonstrated in Figure 5, the system exhibited exceptional specificity for H_2_O_2_, with none of the tested interferents producing significant Δ*A*_652 nm_ responses. These results confirm that radical TTM-DMODPA maintains high selectivity for H_2_O_2_ determination even in complex sample matrices containing multiple potential interferents.

### 3.4. Camera Photography Analysis

**Figure 6:**
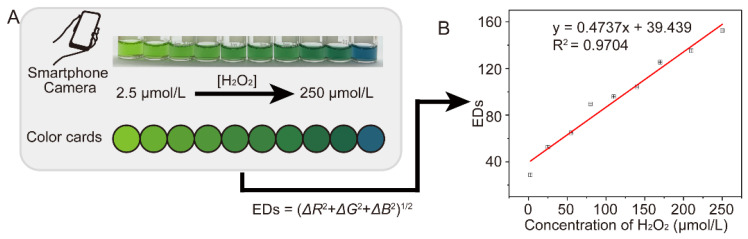
Analysis based on smartphone camera: (**A**) colorimetric picture taken by the camera. (**B**) The linear relationship between EDs value and the added H_2_O_2_ concentration.

To validate the field applicability of our colorimetric assay, we developed a smartphone-based quantitative analysis platform. Following color development at varying H_2_O_2_ concentrations (Figure 6A), solution images were captured using a smartphone camera. Digital image processing was employed to extract RGB color values, which were subsequently converted to Euclidean distances (EDs = (ΔR^2^ + ΔG^2^ + ΔB^2^)^1/2^) for quantitative analysis. The EDs were selected to capture multidimensional hue shifts characteristic of our radical TTM-DMODPA system’s transition from grass green to deep blue, where all three RGB channels exhibit concentration-dependent changes (ΔR = 58%, ΔG = 44%, ΔB = 138%). Most critically, the EDs not only preserve linear correlations across the entire 28.8–152.5 ED range, but also resolve subtle hue variations through their vectorial quantification of triaxial chromatic shifts. This analytical superiority is further corroborated by the dose-dependent response to H_2_O_2_ concentrations (2.5–250 μmol/L) in Figure 6B, where EDs demonstrated exceptional linearity with a calculated detection limit of 3.633 μmol/L (3σ method)—outperforming conventional univariate metrics in both sensitivity and dynamic range coverage. This instrument-free approach combines the analytical performance of laboratory methods with the practicality required for field applications, establishing a robust platform for visual H_2_O_2_ quantification.

### 3.5. Urine Sample Sensing

As demonstrated in previous sections, the radical TTM-DMODPA system exhibits excellent linear response and high selectivity toward H_2_O_2_, demonstrating strong potential for real-sample analysis. Urinary H_2_O_2_ quantification provides valuable clinical information as it serves as a reliable oxidative stress biomarker, an indicator of systemic antioxidant capacity, a screening tool for environmental/occupational hazard exposure, and a potential diagnostic marker for disorders involving aberrant radical metabolism. To evaluate the analytical performance of our system in biological matrices, we conducted standard addition experiments using urine specimens collected from healthy adult volunteers (*n* = 3). In the unspiked urine samples, H_2_O_2_ concentrations were consistently below the method’s limit of detection (LOD: 1.275 μmol/L as specified in Section 3.3), with all measurements yielding signals indistinguishable from the blank controls. Given this undetectable baseline, recovery values were calculated by direct comparison between the measured H_2_O_2_ concentrations in spiked samples and the known amounts of added standard, following the equation: Recovery (%) = (Measured spiked/Added standard) × 100. The yielded recoveries ranged from 96% to 104% across tested concentrations, with all relative standard deviations (RSD) remaining below 4.2% (Table 2). In healthy individuals, urinary H_2_O_2_ concentrations are typically maintained at relatively low levels, while pathological conditions like infections, inflammation, or cancer can significantly elevate these levels through oxidative stress mechanisms [46,60,61,62]. Our optimized colorimetric method, based on the radical TTM-DMODPA, demonstrates excellent translation potential by offering simple, cost-effective detection of pathological H_2_O_2_ changes within the 2.5–250 μM range, suitable for diagnostic applications, treatment monitoring, and personalized medicine approaches, though standardized reference ranges remain important future directions to enhance diagnostic specificity and clinical utility. These robust validation results confirm the method’s reliability for quantitative H_2_O_2_ determination in complex biological samples, particularly for clinical urine analysis applications.

## 4. Conclusions

In conclusion, we have successfully developed a novel colorimetric determination platform based on the radical TTM-DMODPA system, which exploits the specific oxidation of the radical to quinoid oxidation products by H_2_O_2_. The optimized reaction conditions (5 min reaction time, 60 °C, and 150 μmol/L H^+^) demonstrate analytical performance parameters competitive with established methods. Validation studies using human urine samples yielded excellent recovery rates, confirming the method’s reliability for biological sample analysis. The integrated smartphone-based determination platform offers significant advantages for instrument-free, on-site H_2_O_2_ quantification, representing a portable and cost-effective solution for point-of-care testing applications. While this approach provides an innovative tool for biological monitoring and diagnostic purposes, its current implementation is limited to discrete measurements rather than continuous or in situ analysis.

## Figures and Tables

**Table 1:** Comparison of the present work for H_2_O_2_ determination with recently reported methods.

Determination System	Analytical Method	Linear Range (µM)	LOD (µM)	References
N-TiO_2_ + TMB	colorimetric	10–300	2.5	[49]
Co_3_(PO_4_)_2_•8H_2_O + TMB	colorimetric	15–100	4.385	[50]
Fe-doped g-C_3_N_4_ + TMB	colorimetric	2–100	1.8	[51]
Papain + TMB	colorimetric	5–90	2.10	[52]
VB_6_ + TMB	colorimetric	50–600	12.1	[53]
Fe-POF + TMB	colorimetric	5–150	1.8	[54]
CS-Au + TMB	colorimetric	10–400	2.25	[55]
BOD	fluorometric	25–125	4.41	[56]
TZ-BO	fluorometric	0–50	1.0	[57]
N-SiQDs/Ag	fluorometric	5–200	1.5	[58]
Radical TTM-DMODPA	colorimetric	2.5–250	1.275	This work

**Table 2:** Measurements of H_2_O_2_ in human urine samples.

Samples ^1^	Added (µM)	Found (µM)	Recovery (%)	RSD (*n* = 3, %)
1	5.0	4.8	96	4.2
2	10.0	9.8	98	3.3
3	50.0	51.6	104	1.6
4	100.0	102.5	103	2.9

^1^ The urine samples were diluted 10 times for determination.

## Data Availability

The original contributions presented in this study are included in the article/Supplementary Materials. Further inquiries can be directed to the corresponding author.

## Supplementary Materials

The following supporting information can be downloaded at https://www.mdpi.com/article/10.3390/bios15080490/s1, Scheme S1: Synthetic Route for radical TTM-DMODPA; Figures S1 and S2: ^1^H and ^13^C NMR spectra of new compounds; Figure S3: HRMS spectrum (ESI) of radical TTM-DMODPA; Figure S4: Sensing efficiency across the full physiological pH.

## Abbreviations

The following abbreviations are used in this manuscript:

H_2_O_2_	hydrogen peroxide
TTM	tris(2,4,6-trichlorophenyl)methyl
TTM-DMODPA	4,4′-dimethoxydiphenylamine-substituted tris(2,4,6-trichlorophenyl)methyl
HTTM	tris(2,4,6-trichlorophenyl)methane
HTTM-DMODPA	4,4′-dimethoxydiphenylamine-substituted tris(2,4,6-trichlorophenyl)methane
BINAP	2,2′-bis(diphenylphosphino)-1,1′-binaphthalene
*t*-BuONa	sodium tert-butoxide
*t*-BuOK	potassium tert-butoxide
