# Supplementary material for: Oxidation-Active Radical TTM-DMODPA for Catalysis-Free Hydrogen Peroxide Colorimetric Sensing
Source: Biosensors (Basel). 2025 Jul 29;15(8):490. doi: 10.3390/bios15080490 (PMC12384385; doi:10.3390/bios15080490)
Supplement: Supplementary file 1 [file biosensors-15-00490-s001.zip › biosensors-3748152-supplementary.pdf]

Supporting Information

Oxidation-Active Radical TTM-DMODPA for Catalysis-free Hydrogen Peroxide Colorimetric Sensing

Qingmei Zhong*, Xiaomei Rong, Tingting Wu, and Chuan Yan

Hunan Engineering Research Center for Monitoring and Treatment of Heavy Metals Pollution in the Upper Reaches of Xiangjiang River, College of Chemistry and Materials Science, Hengyang Normal University, Hengyang 421008, China
* qingmeizhong2022@163.com; qmzhong@hynu.edu.cn

1. Synthesis of radical TTM-DMODPA.

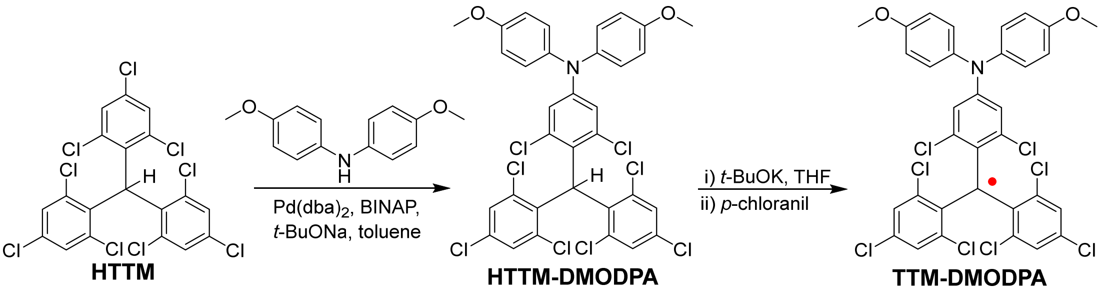

Scheme S1. Synthetic Route for radical **TTM-DMODPA**.

Compound **HTTM** was synthesized according to references.[1]

Synthesis of compound HTTM-DMODPA.

^{1}H NMR (500 MHz, $CDCl_3$) δ (ppm): 7.36 (d, J = 2.5 Hz, 1H), 7.33 (d, J = 2.0 Hz, 1H), 7.25 (d, J = 2.5 Hz, 1H), 7.20 (d, J = 2.5 Hz, 1H), 7.09 - 7.06 (m, 4H), 6.88 - 6.85 (m, 4H), 6.78 (d, J = 2.5 Hz, 1H), 6.65 (d, J = 2.5 Hz, 1H), 6.64 (s, 1H), 3.80 (s, 6H). ^{13}C NMR (125 MHz, $CDCl_3$) δ (ppm): 157.0, 148.9, 139.1, 138.4, 138.0, 137.5, 137.4, 137.2, 137.0, 135.0, 135.0, 133.4, 130.2, 129.9, 128.5, 128.4, 127.7, 124.7, 119.2, 117.8, 115.2, 55.6, 49.7.

Synthesis of radical TTM-DMODPA.

HRMS (ESI, m/z): $[M]^+$ calcd for $C_{33}H_{20}Cl_8NO_2$, 745.8943; found, 745.8936.

The molecular ion peak $[M]^+$ of radical TTM-DMODPA is located at 745.8936 (molecular formula: $C_{33}H_{20}Cl_8NO_2$), and the peaks adjacent to it originate from the isotopic peaks of carbon (^{12}C, ^{13}C) and chlorine (^{35}Cl, ^{37}Cl) atoms in the chemical structure, thus, each mass peaks in Figure 1D can be ascribed to the chemical structure of radical TTM-DMODPA. Here are examples to better understand the generation of these peaks: As shown in Figure S3, all the mass peaks can be divided into three groups, **i)** $[M]^+$ refers to the molecular ion peak, which can be designated as: 33 × 12.0000 (^{12}C) + 20 × 1.0078 + 6 × 34.9689 (^{35}Cl) + 2 × 36.9659 (^{37}Cl) + 14.0031 + 2 × 15.9949 = 745.8941 (found 745.8936); **ii)** *a* group peaks (a_1-a_8) refer to the chlorine change in ^{35}Cl and ^{37}Cl, taking a_1 and a_3 as examples: a_1 can be designated as 33 × 12.0000 (^{12}C) + 20 × 1.0078 + 8 × 34.9689 (^{35}Cl) + 0 × 36.9659 (^{37}Cl) + 14.0031 + 2 × 15.9949 = 741.9001 (found 741.8997); a_3 can be designated as 33 × 12.0000 (^{12}C) + 20 × 1.0078 + 5 × 34.9689 (^{35}Cl) + 3 × 36.9659 (^{37}Cl) + 14.0031 + 2 × 15.9949 = 747.8911 (found 747.8905); **iii)** *b* group peaks (b_1-b_8) refer to the carbon change from ^{12}C to ^{13}C compared with *a* group peaks, taking b_1 as examples: b_1 can be designated as 32 × 12.0000 (^{12}C) + 1 × 13.0034 (^{13}C) + 20 × 1.0078 + 8 × 34.9689 (^{35}Cl) + 0 × 36.9659 (^{37}Cl) + 14.0031 + 2 × 15.9949 = 742.9035 (found 742.9030).

2. Figures S1-S2. ^{1}H and ^{13}C NMR spectra of new compounds.

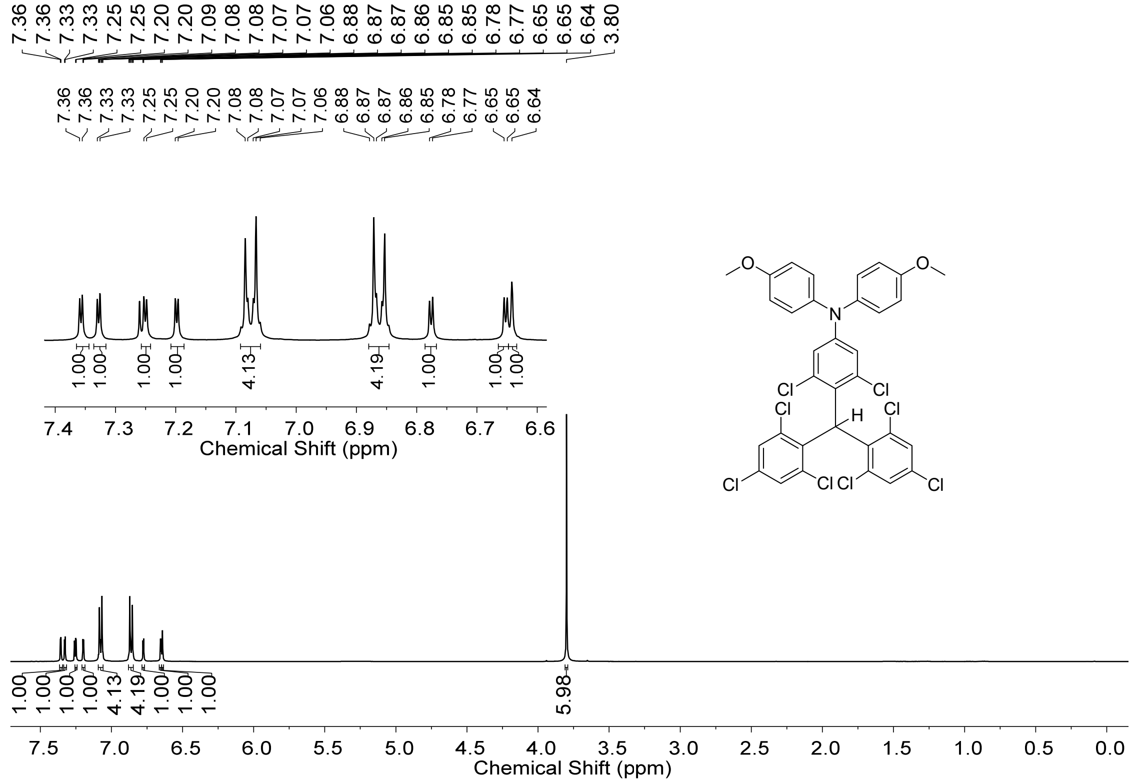

Figure S1. ^{1}**H NMR spectrum of HTTM-DMODPA in CDCl3.**

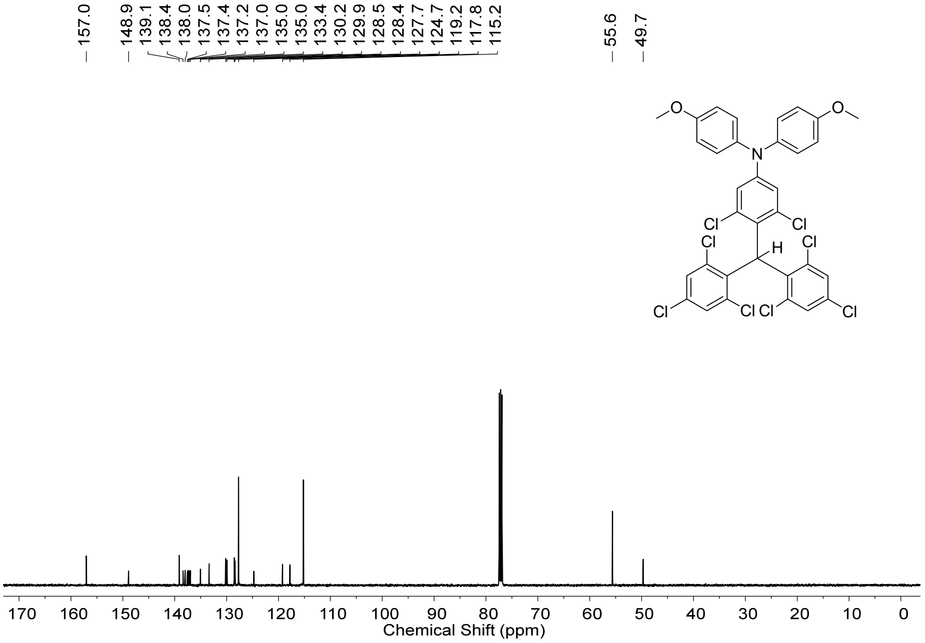

Figure S2. ^{13}**C NMR spectrum of HTTM-DMODPA in CDCl3.**

3. Figures S3. HRMS spectrum (ESI) of radical TTM-DMODPA

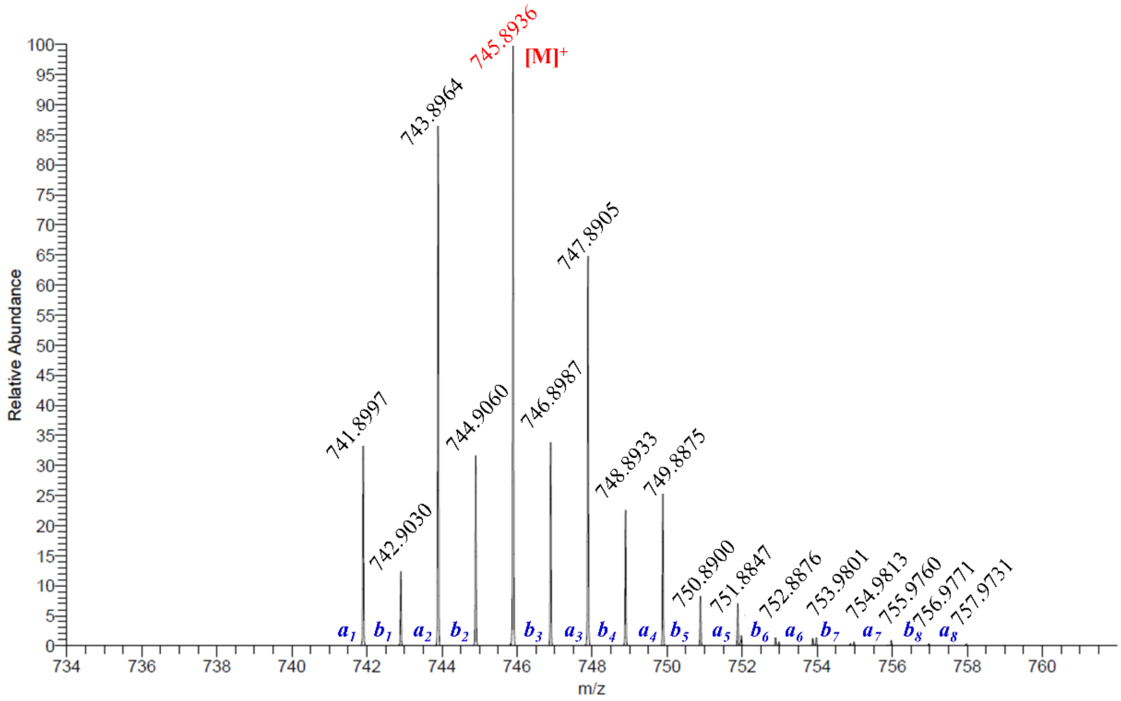

Figure S3. HRMS spectrum (ESI) of radical TTM-DMODPA.

4. Figures S4. Sensing efficiency across the full physiological pH

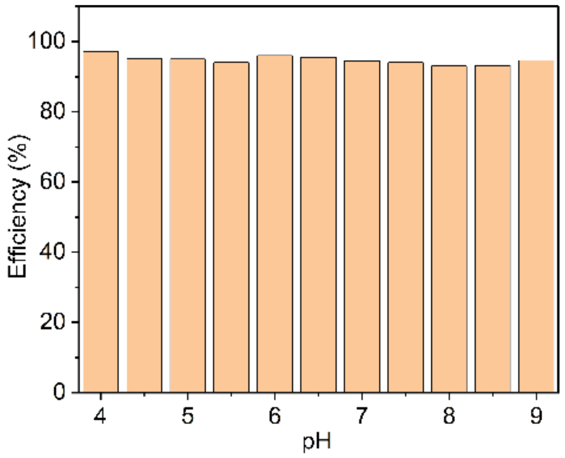

Figure S4. Sensing efficiency across the full physiological pH.

5. References.

1. Peng, Q.; Obolda, A.; Zhang, M.; Li, F. Organic Light-Emitting Diodes Using a Neutral π Radical as Emitter: The Emission from a Doublet. *Angew. Chem. Int. Ed*. **2015**, *54*, 7091–7095.
